# The Impact of Brand Awareness and Country of Origin in the Advertising Effectiveness of Greek Food Products in the United Kingdom: The Case of Greek Yogurt

**DOI:** 10.3390/foods11244019

**Published:** 2022-12-13

**Authors:** Dimitrios Karagiannis, Leonidas Hatzithomas, Thomas Fotiadis, Antonios Gasteratos

**Affiliations:** 1 Department of Business Administration, University of Macedonia, Egnatia 156, GR-546 36 Thessaloniki, Greece; 2 Department of Production and Management Engineering, School of Engineering, Democritus University of Thrace, Vas. Sophias 12, GR-671 32 Xanthi, Greece

**Keywords:** branding, advertising, brand equity, country of origin, Greek yogurt

## Abstract

The present study aims to investigate the country-of-origin effect and the branding process on the brand equity of Greek yogurt abroad, specifically in the United Kingdom. The research was carried out as a 2 × 2 experiment using a closed-ended questionnaire with the participation of a total of 400 consumers, using two Greek yogurts (branded and non-branded) as the product under study, with or without country-of-origin insignia (*viz*. the Greek flag) on the packaging. According to the research results, brand equity was found to be higher for the branded Greek yogurt with country-of-origin insignia among the four product categories, as reflected in its three sub-dimensions (brand awareness, loyalty, and perceived quality). It was also found that country of origin influences brand equity regardless of branding, a finding that confirms the significant effect of this factor on consumers’ perceptions. This highlights the distinction between perceived quality and the specific image of the country in terms of its production experience for a specific product category. Based on these findings, Greek yogurt companies exporting abroad should lay great emphasis on developing branding strategies to strengthen their product brand, while at the same time incorporate country of origin as an indicator of the quality of their brand. The latter finding applies in particular to less well-known brands, since geographical indication (insignia) or other strategies that promote the country of origin are perceived as important informational cues for consumers.

## 1. Introduction

Product advertising depends on a series of factors that shape brand management (branding), the development and adaptation of which are critical activities in modern marketing. Among these factors, product brand and country of origin have emerged as critical variables in shaping consumers’ perceptions, as they directly affect the value of the brand (brand equity) [1], i.e., the set of assets and liabilities associated with a brand, its name and symbolism (logo), which add/or subtract from the value provided by a product or service to the company and/or to its customers [2]. Brand equity is a conceptual and practical construction that has become the subject of an ever-increasing volume of research and practical interest, as it contributes decisively to companies’ profitability, their competitiveness, and the effectiveness of their internationalization strategies [3].

Brand equity is a multidimensional entity that includes several individual dimensions; according to the most popular model to date [2], these are awareness, loyalty, perceived quality, and the connotations that the brand evokes. Though this model has undergone various modifications, it still offers a comprehensive basis for the implementation of research in this field. Several studies have focused, among others, on the factors affecting brand equity, such as the brand itself [4] and its country of origin [5]. In fact, country of origin has emerged as a factor of critical importance for the management of branded products, particularly concerning agri-food goods, for which it has been established as a mark of quality and safety [6]. However, the available research data on dairy products is extremely limited; regarding Greek yogurt in particular, there is no research to date that has studied the factors that shape brand equity for its respective products in foreign markets, despite the intense internationalization and development of exporting activity for this product by domestic companies in recent years.

To this end, the purpose of the paper in hand is to investigate the effect of brand strength and country of origin on consumers’ perceptions of Greek yogurt, as reflected in brand equity and the three individual dimensions (*viz*. awareness, brand loyalty, and perceived quality). To achieve this research purpose, a 2 × 2 experiment was carried out using a questionnaire and two Greek yogurt products (one well known: ‘branded’, and another lesser known: ‘non-branded’), both with or without country-of-origin insignia, namely the Greek flag appearing on the packaging. The research involved 400 consumers in the United Kingdom, a market where Greek yogurt has become extremely popular in recent years. It should be noted that a closed-type questionnaire was used; it evaluates brand equity and the country-of-origin effect, phenomena of major importance for modern marketing and management strategies for the branding of dairy products, especially in terms of their competitiveness in foreign markets. The remainder of this paper is organized as follows: Section 2 provides an overview of the literature regarding brand equity and country of origin (conceptual approach, dimensions, effects, influencing variables, marketing strategies), as well as the relationship between them, both in general terms and in relation to dairy products. Section 3 exhibits the research methodology, where reference is made to the objectives, the research tool, the sample population and the sampling technique, the statistical tools used, and finally the validity and reliability of the results. The research findings are demonstrated in Section 4, through appropriate tabulation, while the last Section is devoted to a discussion of the findings and conclusions, with some limitations and suggestions for future research.

## 2. Literature Review

### 2.1. Brand Equity and Brand Management (Branding)

#### 2.1.1. Conceptual Approach

Brand equity is a concept that has attracted intense research interest since it is regarded as a metric used to gauge the effectiveness of a business’s marketing activities in the frame of its advertising initiatives and product promotions [7]. It is directly linked to organizational performance and the ability of companies to maintain a sustainable competitive advantage over time, attracting more consumers and achieving increased customer loyalty [1]. As stated by Pappu et al. [8], a brand with a high level of brand equity creates positive connotations in the perceptions the consumers have of it, which decisively influences their purchasing decisions. Thus, it is not coincidental that the branding strategies of modern companies, in terms of their marketing activities, focus on increasing the value of the brand [9]. In fact, this trend has been significantly strengthened in recent times, as market globalization and the increased penetration of e-commerce have built up a highly competitive environment, in which a “war” of sorts occurs among popular brands [10].

Theoretically, there are three basic ways to approach the concept of brand equity: from a business, financial, and consumer standpoint. The business approach that bases its success on the effectiveness of its marketing efforts and the value of a brand, which the company derives from various branding procedures, endows the product with added value (e.g., advertising, distribution, promotion) [11]. The financial approach suggests that brands are assets, being subject to trading activities with a specific price; this essentially reflects brand equity, which in turn generates cash flows for the benefit of the enterprise and the acceleration of its development in economic terms [12]. The third approach adopts the perspective of cognitive psychology, which interprets brand equity from the point of view of the consumers themselves, examining the emotional, behavioral, and cognitive connections between them and the brands [13]. Brand equity based on the consumer’s perspective (Customer Based Brand Equity) is one of the most representative indicators for evaluating the effectiveness of modern marketing and companies’ branding activities [1]; it is thus being adopted in the context of the present study.

According to [14], the conceptual definition of brand equity is as follows: brand equity is the added value that a specific brand gives to the corresponding product. Aaker [2], a pioneer researcher in this field, adopts the consumer-based approach, arguing that brand equity is the totality of assets and liabilities associated with a brand, its name and symbolism (logo), which add or subtract from the value provided by a product or service to the business and/or to its customers. In a similar approach, Keller [15] describes brand equity as the differential effect of consumers’ knowledge of a brand in the way they react to that brand’s marketing activities. From the above, it becomes clear that brand equity essentially concerns consumers’ sentiments and perceptions and is related to everything they have learned, felt, been informed, seen, and heard about the brand over time.

#### 2.1.2. The Effect of Brand Equity

Brand equity has been recognized as an extremely important concept in modern marketing, as well as an element of major importance in the management strategies undertaken by companies of branded products; over time, it has been found to decisively affect the performance of a brand, offering a sustainable competitive advantage at a long-term level [16]. According to Molinillo et al. [17], there is an undeniably positive correlation between brand equity and brand loyalty, which translates into increased levels of profitability for businesses through repeat sales, expansion of customer portfolios, and increasing market shares. Sharma [10] argues that brand equity increases the likelihood of the brand’s choice, leading to long-term customer retention, increased willingness to pay for products and services, an upgraded level of quality of communication between consumers and businesses, as well as enhanced profit margins. High levels of brand equity also indicate that the respective companies have the possibility of extending the successful brand name to new product categories, thus diversifying their product portfolio and having access to new sources of competitiveness [18].

Moreover, it has been argued that brand equity reduces the vulnerability of various marketing strategies to changes in the external environment for firms developing their international activities and, at the same time, limiting the elasticity reactions of consumers to corresponding price increases [19]. Furthermore, according to Mahajan et al. [20], brand equity is an important tool for a firm’s geographical expansion and internationalization decisions; it is thus taken very seriously in acquisitions and mergers or other related agreements involving business plans. Similarly, Cifci et al. [21] argue that brand equity constitutes a key mechanism of differentiation between the numerous brands of a product category, affecting its respective performance and providing sources of competitive advantage in international markets. Indeed, recent research has revealed that there is a direct correlation between brand equity and brand performance. For example, in a study by Yang et al. [22] in the e-commerce environment (studying internet search engines), it was confirmed that brand equity positively affects consumers’ satisfaction and loyalty, which are indicators of its performance; Sharma [10] reached similar findings in the electronics industry, studying smartphones.

Furthermore, several studies have focused on the effects of brand equity on various aspects of consumer behavior. In this context, it has been found to positively affect customers’ satisfaction, which in turn produces a positive impact on purchase intention as well as the likelihood of purchasing a different product of the same brand, a concept also known as brand extension [23]. It was also deducted that the higher the recognition of a brand (one of the dimensions of brand equity) the more considerable the willingness to buy or pay a premium for a product of that brand [3].

In a similar study by Rambocas et al. [24], brand equity was also found to lead to an increase in purchase intentions and repeat purchases in the future, building strong brand preferences and reducing consumers’ sensitivity to price increases. In practice, this means that the more strength the company’s brand equity has, the higher the customers’ loyalty. This in turn reduces the cost of marketing activities, increases sales, constitutes a factor in attracting new customers, and creates entry barriers for competitors [25].

#### 2.1.3. Partial Dimensions

Brand equity is a multidimensional concept consisting of various individual dimensions, and each dimension produces different results for businesses [8]. For example, perceived quality enables the consumer to discriminate between products of the same category among different brands, while brand loyalty adds significant value to the firm as it creates a group of customers who remain loyal to the brand for a long period of time and are highly unlikely to switch to a competitor given a lower price [26,27].

Within the literature dealing with the interpretation and understanding of brand equity, two basic approaches can be found for the operationalization of the term based on the individual dimensions. The first approach considers brand equity as an asset of the firm; it enhances the attractiveness of a firm’s products and services, as reflected both in consumers’ perceptions and in its overall value [2]. The second approach assumes that brand equity is built up through consumers’ familiarity with the brand, which results from marketing activities [15]. Although they have different starting points at the theoretical level, these two approaches in essence complement each other and have been used in combination to highlight the respective interpretive models. Among such approaches, the two most popular are those of Keller [15] and Aaker [2].

The first model [15] acknowledges two dimensions: the recognition of the brand and its image. Specifically, recognition refers to the consumer’s ability to recall and identify the brand due to previous exposure to it, while brand image refers to the perceptions a consumer forms through emotional and cognitive connections [15]. Aaker’s model [2] has been used by most of the relevant studies in this field. It suggests that brand equity consists of four dimensions. The first is brand awareness, which refers to the extent a consumer can identify and recall a brand for a specific product category. The second dimension is brand loyalty, which is defined as a deep commitment on the part of the consumer to systematically repurchase a product of a specific brand in the future, thus forming a repeat-purchase behavior, regardless of situational factors or the effects of competitive marketing (by other brands). Therefore, loyalty is associated with a reduced intention to switch to a competing product. The third dimension refers to the connotations a brand is associated with, i.e., representations of what a brand means to a consumer based on own experience. The fourth and final component is the perceived quality, which deals with consumer assessments of a product’s superiority [2]. The Yoo and Donthu revision [28], which is also used in the current study, is an example of this type of modification. It adopts the notion that the awareness factor identifies a brand’s connotations and suggests three elements that go into brand equity: loyalty, awareness, and perceived quality.

As previously mentioned, the model by Aaker [2] has been widely used in the research literature for the development of measurement tools for brand equity, while it has undergone various modifications over time. A typical such modification is the revision suggested by Yoo and Donthu [26], which is also used in the present study. It proposes three factors involved in brand equity: loyalty, awareness, and perceived quality, adopting the notion that a brand’s connotations are identified by the awareness factor.

### 2.2. Country of Origin and Brand Management

#### 2.2.1. Conceptual Approach

Understanding how products’ country of origin affects consumers’ purchasing decisions is an issue that has preoccupied researchers, academics and marketing professionals since the 1960s, when Schooler [29] highlighted this factor as a key influential variable in international trade. In the early years of research towards this direction, emphasis was given to the function of country of origin as an intangible barrier to foreign products entering a new geographic market, given consumers’ natural bias in favor of local products [30]. In the following years, attention was focused on the commercial aspects of the country-of-origin concept, when marketing professionals began to understand that they could use a product’s country of origin as a quality indicator [31]. Today, country of origin continues to attract research interest, given market globalization, the increasing need to differentiate products and services, as well as the increasing costs involved in developing strong commercial logos in the context of international branding [32].

However, even though the country of origin constitutes one of the most popular fields of modern international marketing, it has been argued that this concept has not been sufficiently operationalized, as the theoretical underpinnings are insufficient [33]. The principal idea presented as more theoretically consistent with the concept of country of origin is the country’s image, according to which the ideas, feelings, and connotations associated with a country are directly transferable to the products it produces [34]. Based on this approach, several partial theoretical approaches have been proposed, such as that of Zeugner-Roth and Diamantopoulos [35] who introduced the concept of a difference between the “general” image and the “product” image of a country, pointing out the difference between the consumers’ perceptions of a country in general and its production processes. Josiassen et al. [36] also made a similar distinction for the “origin image” between country, product category, and product.

From the above, it is apparent that product categories comprise a key issue for understanding the concept of country of origin, which has been regarded a significant theoretical concept since the 1980s [37]. Today, it is a common notion that a country’s image varies significantly in consumers’ perceptions depending on the product [38]. For example, a positive image of the country of origin is expected in the case of Italian fashion products, and a negative one is expected in the case of e-government software products from the same country. Therefore, product categories should be taken seriously when assessing the impact of country of origin [39]. It is also important to note that the country of origin for a particular product refers to one or more regions or urban centers, rather than the country as a whole [40]. In the previous example, Italian fashion products refer mainly to the wider area of Milan, which is also considered the world’s fashion capital.

#### 2.2.2. The Effect of Country of Origin

The country of origin is considered an important indicator of consumer behavior, as it creates a decisive effect on the evaluation of products and services, playing a relatively critical role in consumers’ purchasing decisions [41]. As mentioned by Roth and Romeo [42], country of origin is an extrinsic product indicator, i.e., an intangible characteristic of a product, such as price, trademark, and warranty, which is differentiated from other material indicators (e.g., physical presence) being directly related to its performance. In general, it is assumed that consumers are willing to pay more for branded products originating from countries with a positive image [43]. This is explained by the fact that country of origin is often interpreted by the consumer public as a sign of upgraded quality, something which can be exploited during the management of the information load that a consumer bears during the purchasing process [44].

In particular, the country of origin affects some important dimensions of a product’s perceived quality, such as aesthetics, performance, durability, reliability, and functionality [45]. For example, in terms of individual quality dimensions, German cars are considered very reliable, Italian cars more beautiful, and Japanese cars more functional, while in terms of overall quality, French cosmetics, Swiss watches, and Argentinian beef are considered to be of superior quality among the same products with different origins. It has also been established that country of origin significantly affects a brand’s image, which in turn decisively influences purchasing decisions [46]. In the literature, this impact has been described as the effect of the country of origin, a phenomenon directly linked to consumer behavior, the effectiveness of international marketing, and the branding processes of branded products and services [47].

Country of origin has also been examined in relation to the evaluation of foreign (imported) products in the context of international trade [48] and is considered an important competitive factor in the commercialization of products in foreign markets [49]. Previous studies examined the various individual aspects of consumer behavior in relation to the country-of-origin effect. It has been established that positive perceptions of country of origin increase the likelihood of purchasing the respective products [43], in addition to a positive image of the production processes of certain product categories in a country, once again a factor that positively affects the perceptions of the brand strength of the respective products during their evaluation [50].

For example, when consumers first come into contact with a new Australian cheese product, they might have an increased intention to purchase it through the formation of a positive perception of the brand, which derives from the general positive image of Australia (e.g., a developed country), as well as a specific positive image of the production processes involved in the dairy products industry (e.g., production of safe and quality cheeses) [46]. In fact, it has been argued that the country-of-origin effect on consumers’ evaluation of products is also mediated by price, in the sense that when the image of the country is negative or indifferent, then high prices do not have a significant effect, and vice versa [51]. For example, assuming that China and Switzerland have negative and positive reputations respectively for watch production, a high price for a Chinese watch will not affect its perceived quality in a positive way. Conversely, a low price for a Swiss watch is likely to adverse the positive country-of-origin effect, reducing its perceived quality.

The agri-food products market could not remain an exception to the strength of the “country-of-origin effect”. The nation of origin is frequently seen by consumers as the best predictor of the quality [52] or safety [6] of agri-food products, according to pertinent research literature. Moreover, country of origin affects various aspects of consumer behavior, such as intention to pay, preferences, purchase intention, and attitudes [53]. This factor has been investigated in combination with other relevant variables in terms of their cumulative effect, such as a product’s characteristics, the potential for the traceability of its origin, and the brand [54].

These effects have been recorded for various categories of agri-food products. For example, Xie et al. [55] showed that country of origin is a formative factor in the perceived quality of fresh vegetables (broccoli, as mentioned in the study), influencing the corresponding purchase preferences. Hussein and Fraser [56] found that the origin of fresh meat is a notion that is taken seriously by UK consumers when evaluating respective products. Claret et al. [57] also found that country of origin plays a critical role in relation to price in shaping preferences for fresh and frozen fish products, affecting intention to pay, while research by Schjøll [58] showed similar findings for organic products. In relevant research, a conceptual and empirical distinction has also been made between country of origin and the image of the country of origin—these are not always identical—as well as between different product categories. Yeh et al. [59] pointed out, for instance, that the image of the country of origin affects consumer attitudes, whereas Schnettler et al. [60] emphasized considerable variations in the country-of-origin effect for various food products.

Regarding dairy products, evidence from the available research is extremely limited, while for yogurt, which is the focus of the present study, no research was found from a review of the literature. Investigating the segmentation of the corresponding market in Thailand, Unahanandh and Assarut [61] found that country of origin combined with other branding factors (e.g., advertising, nutritional facts, ease of purchase) significantly influenced purchase intention and the perceived premium over price. Schröck [62] also showed that country of origin, as reflected in geographical indications (e.g., P.O.D.), decisively affects consumers’ intention to pay, while Hoang et al. [63] found that a significant influence is also exerted on the purchase intention. Recently, Xu et al. [64] showed that intention to purchase imported dairy products in China directly depends on their country of origin, with the most positive perceptions found for American products: ethnocentrism also emerges as a significant mediating variable in this relationship.

### 2.3. Brand Equity and Country of Origin

From the above analysis, it can be reasonably concluded that country of origin is an important element of the branding process, and it is therefore directly related to brand equity, as examined in relevant studies. For example, Paul and Dasgupta [65] found that positive consumer perceptions of country of origin in the mobile phone industry positively affect the individual dimensions of brand equity, including loyalty, connotations, awareness, and perceived quality. Similar findings were reached by Shahin et al. [5] in the telecommunications product market, calling on marketing managers to implement integrated country of origin promotion strategies.

Yasin et al. [66] focused on the Malaysian home electronics market (televisions, refrigerators, and air-conditioners). They found that country of origin has a positive influence on brand equity, both directly and indirectly, affecting its three individual dimensions (brand visibility, brand loyalty, and brand awareness). Pappu et al. [8] found that brand equity fluctuates according to country of origin, also highlighting the importance of the product category, as this effect was found to be stronger for countries with a long tradition in the production of certain products, a finding that was also confirmed in a later study by the same authors [67]. In other studies, Hamzaoui-Essoussi et al. [68] distinguished between brand origin and country of manufacture (e.g., Apple), showing that, while the former affects all dimensions of brand equity, the latter is only associated with perceived quality. Parkvithee and Miranda [69] also highlighted the importance of consumer involvement as a mediating factor in the relationship between country of origin and brand equity. The studies showed that in cases of low levels of involvement, country of origin plays a limited role in the relevant purchasing decisions, also influencing brand equity to a lesser degree.

In the generic drug industry, Sanyal and Datta [70] found that positive country of origin perceptions increases brand equity overall and in terms of its individual dimensions (brand strength and awareness). Chen et al. [71] confirmed this effect on global industrial markets, using Taiwan as a case study: Taiwan has transformed its production model in recent years by incorporating technological innovations. Moradi & Zarei [72] also found that country of origin plays a mediating role between the positive influence of brand equity on purchase intention and brand preference; this relationship was once again confirmed by Ashill and Sinha [73]. Recently, Kim and Chao [74] highlighted differences between two countries with different production profiles in the technology industry (Korea and China), demonstrating that the image of the country of origin can affect the perceived quality as a dimension of brand equity, but only in the case of China, which is considered to manufacture lower quality technology products compared to Korea.

As in the case of the country-of-origin effect, the available research data for the dairy market on brand equity is very limited. In the few relevant studies available, the multidimensional nature of brand equity has been confirmed, as well as its effect on consumer behavior, including any variables that influence it. For example, Dhanalakshmi and Kohila [75] showed that brand equity for a range of dairy products (cheese, milk, butter) is shaped by five sub-dimensions: brand loyalty, brand image, brand connotations, brand awareness, and the perceived quality of the brand. Accordingly, Drabjerdi et al. [76] confirmed the validity of Aaker’s [2] model regarding the composition of a brand’s value; they found that it is also influenced by other factors related to brand management (packaging, price, advertising, promotional actions, range of distribution). Using a similar approach, Emami [77] showed that brand equity for dairy products is affected by the marketing mix, especially in terms of price and distribution elements, as well as the image of the production company, with high levels of brand equity leading to brand loyalty, brand awareness, and upgraded perceived quality.

The effects of brand equity on consumer behavior in the dairy industry were also the subject of research by Prajapati and Makwana [78], who showed that the purchase intention in this market directly depends on its individual components (awareness, perceived quality, connotations, loyalty). On the contrary, Osman and Subhani [79] found that the intention to buy packaged milk is not significantly affected by brand value, highlighting as a more important factor of this non-correlation the consumer’s low involvement in the purchasing process of this product. It is worth noting that similar findings about the possible influence of consumers’ involvement in the purchasing process have been made in the case of the country-of-origin effect [80].

Similarly, in a study by Taglioni et al. [81], it was found that consumers form their preferences for milk products according to the brand equity they perceive, confirming the relationship between the brand’s value and consumer behavior. An important finding of this study was that consumers tend to place more value on local milk brands, especially those produced by local cooperatives, a finding that indicates a possible relationship between country of origin and brand value. Finally, in the only research that has directly examined the relationship between the country-of-origin effect and brand equity in the dairy industry, Yang et al. [82] found that consumers’ image of different countries, national stereotypes, consumer ethnocentrism, familiarity with the product, and the degree of involvement in the purchasing process are factors that influence the magnitude of the country-of-origin effect. Subsequently, this effect has a direct impact on the individual dimensions of the product’s brand equity (perceived quality, awareness, connotations, and loyalty to the brand). It is worth mentioning that no study to date has examined any effects of country of origin or any other dimensions of brand management in the case of yogurt exclusively, and Greek yogurt in particular.

### 2.4. The Case of Greek Yogurt

Greek yogurt is considered one of the “success stories” of Greek domestic production: through export activity and other internationalization strategies, it has dominated consumers’ preferences in many foreign markets, including the USA and Europe [83]. Greece has a long tradition of yogurt production, which is considered part of its heritage, and it is also recognized internationally [84]. With USD 272 million in exports of yogurt in 2021, Greece ranked third among all exporters. Compared to other countries, yogurt accounted for a substantial share of Greece’s total exports (0.577%) [85]. As one of the oldest foods in the world, yogurt has a high nutritional value, even though it is a low-calorie food, containing various proteins and vitamins, calcium, magnesium, and other elements that have significant benefits for health and good functioning of the digestive system [86]. Greek yogurt has become extremely popular, particularly due to its special composition: compared to other competitive products, it contains almost twice the number of proteins and fewer carbohydrates, while maintaining a pleasant taste and velvety texture [87]. It is therefore no coincidence that Greek yogurt is one of the most recognizable products worldwide [88], with large domestic dairy businesses investing huge sums to strengthen both their production capacity and their internationalization.

Even though Greek yogurt has indisputably conquered foreign markets, most notably the Italian, American, and British markets, research on the marketing activities of Greek dairy companies is extremely limited, both from an operational scope and also from the perspective of the consumers themselves. More specifically, a lack of empirical data is recorded regarding the branding process and its individual elements, with relevant research data found sporadically, a typical example being the study of Desai et al. [89] who examined the sensory properties of Greek yogurt in relation to consumer preferences. In this context, the investigation that follows partially covers this research gap, while adding knowledge and information regarding the value of the brand and the country-of-origin effect in the agri-food products industry. Also, our study contributes to understanding branding in functional foods such as Greek yogurt: products having health-promoting properties. Functional foods are a flourishing area of research [90,91,92], and it is crucial to extend this field.

### 2.5. Research Hypothesis

Based on the above theoretical framework and with the purpose of this study in mind, the following research hypothesis has been formulated:

**H1.**  *The influence of country-of-origin insignia on the overall perceived brand equity is greater for branded products compared to non-branded products.*

## 3. Research Methodology

### 3.1. Research Process

The present study aims to investigate the effect of product brand and country of origin on brand equity, as perceived by consumers of a foreign country, more specifically the United Kingdom. The study also investigates the interaction between the brand and the country-of-origin insignia (the Greek flag) on the packaging, as well as the connotations between the determinant factors involved in the brand value and the importance of country of origin. The research uses a quantitative approach, so a closed-type questionnaire has been developed for its application. This involves a 2 × 2 experiment using images/print advertisements and a questionnaire, the results of which demonstrate the effect of brand awareness and country of origin on the effectiveness of the promotion of Greek food products in the United Kingdom, specifically Greek yogurt, given the positive image of the country in terms of the production of this product category.

### 3.2. Research Tools

The images of different products were chosen as a means of studying the research objectives; for this purpose, the following 4 questionnaires were drawn up:

1. A questionnaire with a photograph of the FAGE Total 2% yogurt packaging, without the Greek flag (a branded product with a well-known brand name in the United Kingdom).
2. A questionnaire with a photo of the DELTA Complet 2% yogurt packaging, without the Greek flag (a product with a weak brand name in the UK).
3. A questionnaire a photograph of the FAGE Total 2% yogurt packaging, with the Greek flag on it (a branded product with a well-known UK brand name, with country-of-origin insignia).
4. A questionnaire a photo of the DELTA Complet 2% yogurt packaging, with the Greek flag on it (a product with a weak brand name in the UK, with country-of-origin insignia).

The participants were assigned randomly to the four treatment groups. Τo conduct the specific research, a closed-type questionnaire was drawn up, consisting of 4 sections. The first section of the questionnaire contained 7 questions to gather data on the demographic characteristics of the sample. All the variables are nominal and concern gender, age, educational level, employment status, profession, annual income, marital status of the respondents, and the number of people their household is made up of.

Before the sample moved on to the second section of the questionnaire, an image of the product packaging with/without the brand marking and with/without the country-of-origin insignia was shown each time. The image was followed by the questions, which were based on the corresponding research tool of Yoo and Donthu [26] concerning *Overall Brand Equity*. More specifically, for this section, consumers were asked to answer if they have ever bought the specific product and if they make systematic use of it with a simple Yes/No response. Following this, the next question referred to the degree to which the respondents intend to buy the product, through a 6-point Likert scale (1—Definitely not to 6—Definitely yes). The section concluded with a question regarding the evaluation of the product in terms of its appearance through a 5-point Likert scale (1—Extremely unattractive to 5—Extremely attractive).

The third section of the questionnaire contained 14 statements based on the Multidimensional Consumer-Based Brand Equity scale by Yoo and Donthu [26]. Respondents were asked to indicate their degree of agreement or disagreement with the respective statements using a 5-point Likert scale (1—Completely disagree to 5—Completely agree). The scale included 4 dimensions that refer to consumers’ perceptions of the product, more specifically: (1) brand loyalty (3 items), (2) perceived quality (2 items), (3) knowledge (awareness) of the brand (5 items) and (4) brand value (4 items). The specific scale does not yield the overall average score, and the results are expressed through the overall average scores on each dimension.

Finally, the fourth section of the research tool includes 10 statements developed on the basis of the theoretical background of Roth and Romeo [42]. Once again, respondents were asked to indicate their degree of agreement or disagreement with the respective statements using a 5-point Likert scale (1—Completely disagree to 5—Completely agree). Through the calculation of the overall average rating, a dimension is obtained that determines the effect of the country-of-origin insignia for the product contained in the presented packaging.

### 3.3. Sample and Sampling Procedure

The complete study sample amounted to 400 people. The sampling method used was convenience sampling, which is a type of non-probability sampling. Based on this method, the sample consisted of individuals who were selected from the population according to the criteria of their availability and their willingness to participate in the research [93] (Elliot and Haviland, 2007), with the potential research population consisting of all consumers of the United Kingdom. The survey was conducted electronically, developed on the online Google forms platform. Subsequently, the electronic platform *figure-eight.com* was used to recruit the sample, with the responses being entered directly into Google forms spreadsheets. Sampling began in December 2019, and the desired number of 100 completed questionnaires for each of the 4 different questionnaire types was reached in February 2020.

Table 1 shows that 68.0% of the sample were men and 32.0% were women, while 24.0% were 18 to 24 years old, 22.5% were 25 to 29 years old, 27.5% were 30 to 39 years old, 13.5% were 40 to 49 years old, 11.5% were aged 50 to 59, and 1.0% were over 60 years of age. Concerning educational level, 12.2% of the survey participants had completed only primary education, 24.5% secondary education, and 11.8% vocational education, while 44.8% were tertiary education graduates and 6.8% were postgraduates or held a doctoral degree. Regarding respondents’ occupations, 14.8% were public employees, 45.5% private employees, 16.8% students, 13.5% freelancers, 5.2% unemployed, and 4.5% dealt with domestic work. 13.5% of respondents earned an annual income of up to £15,000, 24.2% £15,001–25,000, 34.5% £25,001–35,000, 16.8% £35,001–50,000, 3.8% £ 50,000–70,000, and 7.2% over £70,001. Concerning marital status, 40.0% of the respondents were single, 46.0% married, 8.5% divorced, 4.5% separated, and 1.0% widowed. Finally, concerning the number of people living in their household, 18.8% answered that their household consisted of 1 person, 19.8% lived in a 2-person household, 40.8% with 3–4 people, 14.0% with 5–6 people, and finally, 6.8% had more than 7 people living in the same household.

### 3.4. Statistical Tools

To capture the research results, based on the goals that had been identified, both descriptive and inductive statistical tools were used. More specifically, tables showing absolute and relative frequencies were utilized to present the demographic data of the sample. These tools were also used to study consumers’ overall behavior and their perceptions towards the presented products. The parametric t-test for independent samples was used to compare the mean ratings of the dimensions of Brand Loyalty, Perceived Quality, Brand Knowledge, Brand Value, and Country of Origin based on the presence or absence of country-of-origin insignia and that of the logo (brand) on the product packaging. Furthermore, for the comparison of the average ratings of the above dimensions, based on the overall characteristics of the products (presence of country-of-origin insignia and brand strength), the parametric One-way Analysis of Variance Test is also used for the control of statistically significant differences in the average pairwise ratings of the products (Bonferonni’s post-hoc test). Finally, to investigate the correlation of the presented pairwise dimensions, Pearson’s correlation test (α = 5%) is used.

### 3.5. Validity and Reliability

In the context of the planning and execution of this research, every possible effort was made to ensure that it met the necessary conditions of validity and reliability. Before the implementation of the actual sampling, a pilot survey was conducted on a sample of 5 people, in order to establish that the questionnaire can be answered without problems. During the sampling process and before completing the questionnaire, respondents were also asked for their consent to participate in the survey, without revealing the subject or its goals. It was also clarified to them that the collected data will remain protected by the researchers and that the individuals’ personal information will not be stored and/or used for any other purpose. Finally, respondents were clearly informed that they are free to withdraw from the research at any time, highlighting that their agreement to participate in the research will be valid as long as the resulting information will remain anonymous. Finally, to ensure that the data used in the study showed high internal reliability, Cronbach’s Alpha coefficient was extracted, which showed a satisfactory internal consistency of the data for all samples and dimensions, since it was higher than 0.6 in each case.

## 4. Research Results

Table 2 presents the results regarding consumers’ overall behavior and perceptions of the products in the images shown to them, based on whether the products are branded or not. It was initially observed that the percentage of consumers who have purchased the branded product is significantly higher in relation to the percentage of those who have bought the non-branded product, with similar results regarding the systematic use of the two products by the consumers. At the same time, there is a high degree of purchase intention for the branded product, as 55.0% of the respondents have a positive attitude towards it, with the corresponding percentage for the non-branded product being equal to 23.5%. Moreover, the percentage of respondents who consider the branded product attractive or extremely attractive is also very high (61.0%), while 23.0% consider it neutral, and 16.0% think it is unattractive or extremely unattractive, with percentages for the non-branded product being equal to 29.5%, 46.0 and 24.5% respectively.

Table 3 shows the results of the One-Way Analysis of Variance test to compare the mean scores of the dimensions of the logo (brand) and the branding impact, based on country-of-origin insignia (the Greek flag) and the logo (brand) marking; the individual differences in the mean scores generally proved to be statistically significant (*p* < 0.001). Consumers show a particularly high level of loyalty to the branded product with country-of-origin insignia, in contrast to loyalty to the branded product without country-of-origin insignia and to the non-branded product with country-of-origin insignia, which is lower than average. At the same time, respondents’ loyalty to the non-branded product without country-of-origin insignia is particularly low. In relation to perceived quality, it is actually quite high for the branded product with country-of-origin insignia, and also high for the branded product without country-of-origin insignia. On the other hand, the perceived quality of the non-branded product with country-of-origin insignia is moderate, whereas for the non-branded product without country-of-origin insignia, it is low. Similar results are obtained regarding brand awareness, which is high for the branded product with country-of-origin insignia, and above average for the branded product without country-of-origin insignia, while consumers show moderate brand awareness of the non-branded product with county of origin insignia and low knowledge of the non-branded product without country-of-origin insignia. Moreover, consumers perceive a high brand value for the branded product with country-of-origin insignia, a relatively high value for the branded product without country-of-origin insignia, a lower than moderate value for the non-branded product with country-of-origin insignia, and an extremely low value for the non-branded product without country-of-origin insignia. Finally, the degree of the effect of country-of-origin insignia on consumers’ perceptions to the branded product with country-of-origin insignia is high, while it is lower for the branded product without country-of-origin insignia, medium for the non-branded product with country-of-origin insignia, and low for the non-branded product without country-of-origin insignia.

In fact, through the respective post-hoc analysis using Bonferonni’s method (Table 4), it can be observed that, in each case, the respondents’ average scores regarding the branded product with country-of-origin insignia are consistently and statistically highly significant, followed by the average scores of the branded product without country-of-origin insignia. Furthermore, the mean ratings of the non-branded product with country-of-origin insignia are more highly consistently and statistically significant than the mean ratings of the non-branded product without country-of-origin insignia. It should be highlighted that in just one single case of pairwise comparisons, no statistically significant differences arose, and this concerns the level of loyalty to both the logo/brand for the branded product without country of origin and the non-branded product with country-of-origin insignia (*p* = 1.000).

## 5. Discussion and Conclusions

### 5.1. Discussion of the Findings

The branding process of dairy products is a value-added activity for modern companies, especially those who are trying to internationalize, a typical example being the Greek yogurt production companies that have recently dominated several foreign markets. In this context, an understanding of the factors that shape consumer perceptions and attitudes towards Greek yogurt products acquires special value at both the research and practical level. The purpose of this work was to carry out a 2 × 2 experiment on a sample of consumers in the United Kingdom, a country ranking in the second place as far as Greek yogurt exports are concerned, in an attempt to investigate whether there are any differences between branded and non-branded products, based on the value of the brand, and also to examine the country-of-origin effect. In the present study, it was found that brand equity is significantly higher for branded products and for products with geographical indications. Indeed, previous research has already shown that brand equity is positively influenced by a product brand and other related variables, such as perceived brand personality, prior experience, and the emotional, cognitive, and sensory relationship that has been developed between the consumer and the brand with relation to the product [4,94,95].

The present findings also agree with previous studies demonstrating that brand equity is significantly affected by some kind of geographical indication, confirming the strength of the country-of-origin effect on the management of branded products (branding) [32,43,46]. In fact, the three sub-dimensions of brand equity (loyalty, awareness, and perceived quality) score higher ratings for branded and geographically labeled yogurt products, compared to the other three product categories, a finding highlighting a possible co-influence of the country origin and a strong brand name, as has been suggested by various studies [5,48,65,82].

Another notable result of the study is that the individual dimensions of brand equity were found to be higher for non-branded products that bear country-of-origin insignia. This finding suggests that products not belonging to a popular brand can benefit strongly by the country-of-origin effect, which can be attributed to the consumer’s belief regarding a product’s upgraded quality and the positive image the consumer may perceive for the country of origin [43,46]. This relationship has also been confirmed in the food industry [6,52]. It is also explained by the fact that, according to consumers’ beliefs, Greece is a pioneer and has a long tradition in the production of quality yogurt, as a country, which confirms the importance of national stereotypes in the interpretation of the country-of-origin effect [35,96]. It is also argued that country of origin is more often used as an indicator for products that consumers have limited knowledge of and familiarity with, such as less well-known brands [97], and also for products with lower consumer involvement in the purchase process [80,98], as is probably the case regarding the purchase of a non-branded yogurt.

The overall findings of the study at hand are that brand equity in Greek yogurt products is higher for branded products with geographical indications, confirming the direct effect of country of origin and the strength of the brand in this perceived value, as has also been found in other studies for a range of product categories [70,72,74]; this has also been confirmed in recent research in the dairy products industry [82]. This study also supports the relationship between positive perceptions of a product and a positive image of the respective country of origin in cases of countries with a relevant tradition in the production of the product [59,60], highlighting the role played by the respective product category [8,67] and also possibly the degree of consumers’ involvement in the purchase process, which depends mainly on the nature of the product [69]. Finally, it can be concluded that brand strength is a critical component in the branding process, as it decisively affects consumers’ perceptions of brand equity [4,99].

### 5.2. Conclusions

The contribution of the present research lies in the identification of important factors that contribute to the branding process involved in Greek yogurt production, focusing on the influence of the brand and the country of origin in the context of brand equity, a conceptual and practical construct that calls for a great deal of research and practical interest in the field of modern marketing. At the theoretical level, the study confirmed the multidimensional nature of brand equity, as proposed within the last few decades by leading theorists and researchers. At the same time, it has also added new knowledge at an empirical level regarding the role played by the country of origin in the branding process of Greek yogurt, which is important for both branded and non-branded products. It can therefore be argued that the country’s long tradition of producing quality yogurt with distinct characteristics, in comparison to competitors’ products, translates into positive perceptions of the foreign market’s consumers for the respective products, regardless of the brand name. At the same time, it becomes clear that the combined influence of a strong brand and a positive image of the country of origin leads to the formation of very strong brand equity, as this can be understood by the highest levels of brand loyalty, perceived quality, and awareness. Therefore, it is an indisputable fact that outside its country of origin, a strong brand constitutes a variable of enhanced perceived brand value from the consumers’ point of view.

On a practical level, the findings of this study could also be used by marketing professionals dealing with the further promotion in foreign markets of Greek dairy products, especially yogurt. Initially, upgrading the perceived quality of the individual elements of Greek yogurt branding (e.g., packaging, composition, differentiation, taste, caloric and nutritional value, distribution, and positioning compared to competitors’ products) can contribute to increasing brand equity for both branded and non-branded products, leading to enhanced positive consumers’ perceptions, thus, increased long-term loyalty and repeat purchases. Secondly, the promotion of the country of origin can similarly lead to increased sales and greater profitability for Greek dairy companies operating in foreign markets, even though the most appropriate branding strategy for each product or market should be selected and implemented, e.g., use of P.D.O. labels in the European area, or endorsement by well-known personalities connected to Greece, e.g., using a celebrity to advertise the product in the USA market. Finally, the findings of the present study confirm that promoting a product’s country of origin is more important for less well-known yogurt brands, as this factor constitutes an indicator of the product’s quality used by consumers regardless of brand strength, a fact that should be taken into account by marketers of Greek businesses.

### 5.3. Limitations and Suggestions for Future Research

The present study is characterized by a number of limitations that should be well noted. The first is the relatively limited sample, at least in geographical terms, as all the research participants resided exclusively in the UK, which may raise issues regarding the generalizations of the findings. Secondly, the research is limited by the fact that it does not attempt to examine other factors, which may directly or indirectly play some role in the relationship between brand equity and country of origin, such as national stereotypes, ethnocentrism, and consumer involvement in the purchasing process. Another limitation of the study is that only one product category was examined, since that particular product is a key variable for investigating the influence of country of origin in its marketing. Finally, it should be noted that the Greek yogurt brand used in the study has been on the UK market for more years than the lesser-known brand; it may thus be the case that the consumers who took part in the study were biased in its favor to some extent.

According to the above, future research concerning the branding process of Greek yogurt should further examine the effect of country of origin and brand on brand equity, in relation to the latter within the frame of consumer behavior. This kind of study in particular should also investigate whether and to what extent country of origin and brand influence consumer initiatives and purchasing habits, as well as loyalty and intention to purchase, pay for, and recommend a product to third parties. Of particular importance for future research is the use of larger samples from more countries around the world where Greek yogurt is popular (e.g., USA, Italy), in order to investigate geographical differences and whether these do in fact exist. Finally, the inclusion of other influencing factors, such as consumers’ involvement in the purchase process, national stereotypes, and ethnocentrism should also be the subject to future research in this field. The latter (ethnocentrism) was of particular research interest in terms of the state of the UK market in the post-Brexit era, given the effects of Brexit on the free movement of products in the context of the laws and regulations of the common European market.

## Figures and Tables

**Table 1:** Demographic elements.

		*N*	%
**Gender**	Male	272	68.0%
	Female	128	32.0%
**Age**	18–24	96	24.0%
	25–29	90	22.5%
	30–39	110	27.5%
	40–49	54	13.5%
	50–59	46	11.5%
	Over 60	4	1.0%
**Educational level**	Primary education	49	12.2%
	Secondary education	98	24.5%
	Vocational education	47	11.8%
	Tertiary education	179	44.8%
	Post-graduate and doctoral studies	27	6.8%
**Occupation**	Public employee	59	14.8%
	Private employee	182	45.5%
	Student	67	16.8%
	Freelancer	53	13.2%
	Unemployment	21	5.2%
	Domestic work	18	4.5%
**Annual income**	up to £15,000	54	13.5%
	£15,001–25,000	97	24.2%
	£25,001–35,000	138	34.5%
	£35,001–50,000	67	16.8%
	£50,001–70,000	15	3.8%
	over £70,001	29	7.2%
**Family status**	Unmarried	160	40.0%
	Married,	184	46.0%
	Divorced	34	8.5%
	Separated	18	4.5%
	Widowed	4	1.0%
**Number of people in the household**	1	75	18.8%
	2	79	19.8%
	3–4	163	40.8%
	5–6	56	14.0%
	over 7	27	6.8%

**Table 2:** Overall behavior and perceptions of consumers towards products based on brand.

		Brand			
		Branded Product		Non-Branded Product	
		N	%	N	%
**Have you ever bought this product?**	Yes	112	56.0%	65	32.5%
	No	88	44.0%	135	67.5%
**Do you use this product?**	Yes	85	42.5%	48	24.0%
	No	115	57.5%	152	76.0%
**Would you like/Do you intend to buy this product?**	Absolutely not	16	8.0%	32	16.0%
	Probably not	31	15.5%	45	22.5%
	Possibly not	43	21.5%	76	38.0%
	Possibly	42	21.0%	24	12.0%
	Probably	42	21.0%	13	6.5%
	Absolutely	26	13.0%	10	5.0%
**How would you judge this product?**	Exceptionally unattractive	15	7.5%	24	12.0%
	Unattractive	17	8.5%	25	12.5%
	Neutral	46	23.0%	92	46.0%
	Attractive	39	19.5%	26	13.0%
	Exceptionally attractive	83	41.5%	33	16.5%

**Table 3:** Results of the One-Way Analysis of Variance test.

Product Features											
	Branded Product without Country-of-Origin Insignia		Branded Product with Country-of-Origin Insignia		Non-Branded Product without Country-of-Origin Insignia		Non-Branded Product with Country-of-Origin Insignia				
	M.O.	T.A.	M.O.	T.A.	M.O.	T.A.	M.O.	T.A.	F	B.E.	*p*
**Loyalty to the logo/brand,**	2.79	0.93	3.81	0.86	1.65	0.65	2.68	0.78	117,488	399	0.000
**Perceived quality**	3.60	0.64	4.43	0.47	2.23	0.94	3.16	0.63	173,970	399	0.000
**Knowledge of the logo/brand,**	3.37	0.53	4.22	0.57	2.15	0.77	3.05	0.61	185,754	399	0.000
**Value of the commercial logo/brand,**	3.40	0.61	4.01	0.66	1.97	0.80	2.85	0.62	164,436	399	0.000
**Effect of country-of-origin insignia**	3.33	0.51	4.23	0.49	2.57	0.83	3.08	0.47	136,824	399	0.000

**Table 4:** Post-hoc analysis.

	Loyalty to the Logo/Brand		Perceived Quality		Knowledge of the Logo/Brand		Value of the Commercial Logo/Brand		Effect of Country-of-Origin Insignia	
	M.A.	*p*	M.A.	*p*	M.A.	*p*	M.A.	*p*	M.A.	*p*
Branded product without country-of-origin insignia- Branded product with country-of-origin insignia	−1.01	0.000	−0.83	0.000	−0.85	0.000	−0.61	0.000	−0.90	0.000
Branded product without country-of-origin insignia- non-branded product without country-of-origin insignia	1.14	0.000	1.37	0.000	1.22	0.000	1.43	0.000	0.76	0.000
Branded product without country-of-origin insignia-non-branded product with country-of-origin insignia	0.11	1.000	0.44	0.000	0.32	0.002	0.56	0.000	0.24	0.024
Branded product with country-of-origin insignia- non-branded product without country-of-origin insignia	2.15	0.000	2.20	0.000	2.07	0.000	2.04	0.000	1.67	0.000
Branded product with country-of-origin insignia- non-branded product with country-of-origin insignia	1.12	0.000	1.27	0.000	1.17	0.000	1.16	0.000	1.15	0.000
Non-branded product without country-of-origin insignia- non-branded product with country-of-origin insignia	−1.03	0.000	−0.93	0.000	−0.90	0.000	−0.88	0.000	−0.52	0.000

## Data Availability

The data presented in this study are available on request from the corresponding author.
